# Impact of Dominant Species Shift in Herbaceous Vegetation Beneath Sand-Fixing Plantations on Soil Microbial Communities Involved in Organic P Mineralization and Inorganic P Solubilization

**DOI:** 10.3390/plants15142175

**Published:** 2026-07-15

**Authors:** Herui Li, Ying Zhang, Yang Xiang, Chengyou Cao

**Affiliations:** 1 College of Life and Health Sciences, Northeastern University, Shenyang 110169, China; lihetui-neu@hotmail.com (H.L.); zhangying@mail.neu.edu.cn (Y.Z.); xiangyang-2025@hotmail.com (Y.X.); 2 Liaoning Province Key Laboratory of Bioresource Research and Development, Northeastern University, Shenyang 110169, China

**Keywords:** vegetation succession, *phoD* gene, *gcd* gene, plant–soil feedback, sand-fixing plantation

## Abstract

Herbaceous vegetation under sand-fixing plantations is critical for ecosystem stability. Phosphorus (P) represents a key growth-limiting factor for plants in sandy soils, yet systematic understanding of P-cycling microbial communities including their composition, ecological functions, and responses to vegetation succession remains limited. We conducted cross-transplant experiments using dominant species from different successional stages under *Caragana microphylla* plantations in the Horqin Sandy Land, and assessed soil properties, enzyme activities, organic P mineralization and inorganic P solubilization rates, and *phoD*/*gcd*-harboring microbial communities. Encroachment by late-successional species increased soil nutrients, biological activities, *phoD*/*gcd*-microbial abundances, and soil P transformation rate, and restructured microbial communities while preserving core taxa. Soil properties (pH, electrical conductivity, organic matter, total nitrogen, total P, available potassium, available P, and NH_4_^+^-N) significantly influenced *phoD* community composition. Notably, P availability was primarily driven by *phoD*-mediated organic P mineralization, with *gcd*-mediated solubilization playing a minor role. These changes enhanced alkaline phosphatase activity and P availability, promoting plant growth and new species colonization, thereby establishing a positive plant–soil–microbe feedback that drives succession. The identified dominant *phoD*/*gcd* taxa provide candidates for developing microbial inoculants to accelerate vegetation restoration.

## 1. Introduction

Land desertification, driven primarily by climate change and unsustainable human activities, represents the most significant ecological challenge in arid, semi-arid, and dry sub-humid regions [1]. The Horqin Sandy Land, encompassing approximately 51,800 km^2^ in western Northeast China, historically supported a forest-steppe ecosystem characterized by abundant lakes, dense forests, and grasslands, serving as a traditional pastoral area. Over the past century, however, widespread grassland degradation into sandy land driven by overgrazing and arid climatic conditions has rendered this region one of China’s most severely desertified zones. Vegetation restoration and reconstruction constitute the primary strategy and fundamental task for comprehensively rehabilitating degraded ecosystems in the Horqin Sandy Land. In recent decades, large-scale sand-fixing vegetation comprising trees, shrubs, and semi-shrubs has been established artificially on mobile and semi-mobile dunes. These plantations play a critical role in mitigating wind and sand hazards [2]. When establishing sand-fixing vegetation, while wind prevention, sand fixation, and ecological improvement are primary objectives, equal consideration must also be given to the vegetation’s own development and the sustainability of these functions. This latter aspect, however, has long been overlooked and lacks comprehensive systematic research. Positive plant–soil feedback can promote the gradual formation of complex and stable community structures. Without these conditions, the windbreak and sand-fixation capacity of this vegetation will decline significantly. Artificially established shrub-based sand-fixing forests typically stabilize sand surfaces and modify the micro-environment within 3–5 years, facilitating the colonization of herbaceous plants. Consequently, the formation and succession of the understory herbaceous plant community progress alongside the overall development of the sand-fixing ecosystem. Understory herbaceous vegetation plays a vital role in maintaining the function and stability of sandy land ecosystems. It contributes to stabilizing the sand surface and conserving soil and water. Moreover, its substantial annual litterfall provides a significant source of soil organic matter, fostering rapid microbial proliferation and thereby accelerating soil nutrient cycling processes. The role of herbaceous vegetation is particularly prominent in the restoration and reconstruction of secondary bare lands, such as mobile dunes. Crucially, once a community dominated by perennial herbaceous plants establishes itself, it can sustain its windbreak and sand fixation functions even in the event of degradation within the sand-fixing plantation.

The fundamental drivers of understory herbaceous plant community succession originate from within the community itself, primarily via interactions among different populations, between plants and the soil environment, and between plants and animals/microorganisms. Central to these complex interactions is the plant–soil microorganism feedback. This feedback mechanism influences soil physicochemical properties, biological activity, and the availability of water and nutrients, thereby altering the interrelationships among ecological factors within the plant community. Hence, the modification of plant behavioral processes ultimately results in continuous changes in community structure and function [3,4,5,6]. However, the specific processes and mechanisms by which these plant–soil microorganism feedback interactions drive community succession remain unclear. Furthermore, the synergistic effects among these three components require further exploration and refinement, and the response patterns of plant, soil, and microbial communities to this synergy are still poorly understood. Therefore, a deep analysis of the feedback relationships among these three elements is crucial for elucidating the intrinsic mechanisms underlying plant community formation and succession.

Community succession involves the continuous disruption of old equilibria and the establishment of new ones. The replacement of dominant species represents the most conspicuous characteristic of this process. Consequently, investigating the biological mechanisms underlying species replacement is a prerequisite and foundation for a deeper understanding of succession dynamics. Plant–soil microorganism feedback interactions can alter interrelationships among various biological and environmental components within the community, thereby regulating numerous ecological processes. These include individual plant growth, biodiversity, community structure, nutrient allocation and cycling, and the formation of plant-microbe symbioses. This critical role has garnered increasing attention from ecologists, establishing feedback interactions as a key focus for research into the driving mechanisms of community succession [7,8].

Plant species exhibit distinct biological and ecological traits. Their soil nutrient demands and growth strategies shift markedly in response to environmental conditions, thus triggering changes in community structure and functional processes. Species colonizing early succession stages are typically fast-growing annuals with high resource acquisition capacity but low tolerance to environmental fluctuations. In contrast, species characteristic of later stages are often slow-growing perennials exhibiting greater tolerance and resilience to environmental disturbances. During community succession, niche differentiation driven by interspecific competition progressively modifies strategies for soil nutrient utilization [9]. In the formation of plant communities, soil physicochemical properties and specific functional microbial groups serve as key drivers. They influence individual plant growth and community biomass, while simultaneously fostering plant-microbe symbioses that enhance plant stress tolerance and alter soil biological activity [10]. The feedback relationships among these three components manifest across multiple scales, encompassing the life history and physiological-biochemical traits of individual plants, ecological functions, as well as community structural dynamics, biomass, and resistance to adverse environmental conditions [11,12].

In most terrestrial ecosystems, phosphorus (P) is a primary limiting factor for primary production alongside nitrogen (N). Furthermore, P is the most difficult nutrient to acquire and utilize in soils. While the total soil P pool is substantial, the concentration of soluble phosphate available for uptake by most organisms is very low. The turnover and transformation of soil P forms are primarily driven by soil microorganisms. Soil microbes enhance P availability through various mechanisms, particularly by promoting the mineralization of organic P and solubilization of inorganic P, thereby increasing nutrient supply to plants and other organisms [13]. These processes are mainly catalyzed by soil phosphatases, enzymes secreted by microorganisms or plant roots. Alkaline phosphomonoesterase (ALP) secreted by soil microorganisms is recognized as a key enzyme for converting soil organic P compounds into plant-available phosphate (PO_4_^3−^) [14,15]. ALP encoding genes can be categorized into three distinct families: *phoA*, *phoD*, and *phoX* [16,17]. Notably, multiple studies confirm a strong positive correlation between *phoD* gene abundance and soil ALP activity [18,19,20]. Therefore, *phoD* can serve as a key molecular marker for analyzing microbial communities involved in soil P cycling. Its abundance effectively indicates both soil ALP activity and the abundance of organic P-mineralizing bacteria [13,21]. Phosphate solubilization, predominantly mediated by phosphate-solubilizing bacteria (PSB), constitutes another major source of bioavailable soil P. PSB secrete organic acids that lower soil pH and chelate insoluble P compounds, converting them into soluble forms. Research indicates that microbial phosphate-solubilizing activity depends on the *gcd* gene, which encodes quinoprotein glucose dehydrogenase. Crucially, in PSB, the *gcd* gene governs gluconic acid production and mineral phosphate solubilization [22,23]. This demonstrates that PSB carrying the *gcd* gene play a significant role in enhancing soil P bioavailability.

This study investigates herbaceous vegetation beneath *Caragana microphylla* sand-fixing plantation in the Horqin Sandy Land. Our objectives are to: (1) examine how shifts in dominant herbaceous species during succession impact soil physicochemical properties and microbial activity; (2) characterize responses of soil microbial communities (specifically *phoD*- and *gcd*-harboring populations) to species replacement in terms of abundance and community structure; and (3) identify key drivers of soil P availability throughout vegetation succession. We hypothesize that the invasion of the dominant species from a later successional stage into the soil of an earlier stage significantly alters soil properties and biological activity, affects the abundance and community structure of *phoD*- and *gcd*-harboring microorganisms, and thereby drives the successional process through changes in plant–soil interactions. These findings are expected to establish a theoretical foundation for evaluating ecological benefits, optimizing structural configuration, and predicting long-term development trajectories of sand-fixing vegetation.

## 2. Results

### 2.1. Impact of Late-Successional Dominant Species Encroachment on Soil Properties

The measured values of pH, electrical conductivity (EC), organic matter (OM), total N (TN), total P (TP), total K (TK), ammonium N (NH_4_^+^-N), available P (AP), and available K (AK) for soil samples under different treatments are presented in Table 1. Overall, encroachment by subsequent-stage dominants significantly altered most soil properties, but the magnitude and direction of change were species- and stage- dependent. The most pronounced modifications occurred during BD encroachment onto MSD, where EC, OM, AP, and AK increased markedly (1.43- to 2.14-fold for EC, OM, and AP, and 1.69-fold for AK), while TN, TP, and NH_4_^+^-N decreased significantly. In contrast, CA encroachment onto ESS mainly affected EC, NH_4_^+^-N, AP, and AK (with EC declining, and NH_4_^+^-N and AP rising), leaving pH, OM, TN, TP, and TK unchanged. For CS encroachment onto MSS, significant changes were observed for most indices except TP and TK; pH, EC, NH_4_^+^-N, AP, and AK decreased, whereas OM, TN, and TP increased. These results demonstrate that the impact of encroachment on soil properties is not uniform across successional stages: early-stage invasion (BD → MSD) exerted the strongest effects, while later-stage invasions produced more selective or contrasting changes, suggesting complex feedbacks between plant turnover and soil fertility.

**Figure 1:**
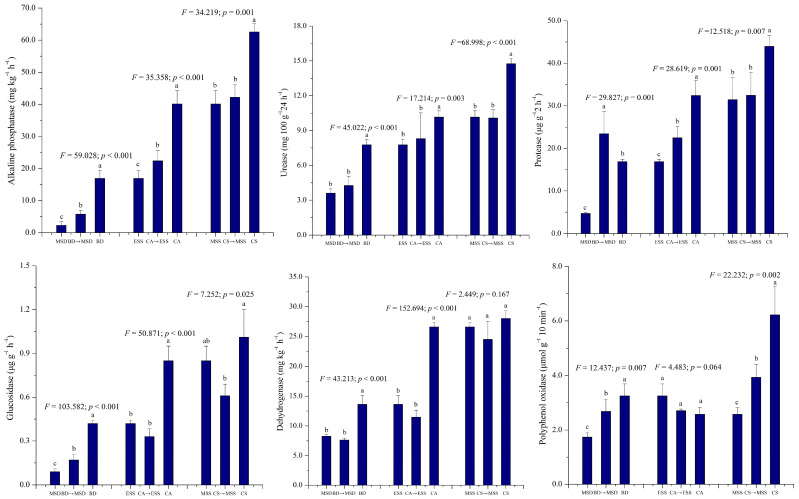
Effects of encroachment by later successional dominant species on soil enzyme activity of the preceding stage. Values are means ± SD. *F* and *P* values are derived from one-way ANOVA. Means sharing the same lowercase letter are not significantly different (*p* < 0.05). Abbreviations: MSD, mobile sand dune; ESS, early-successional stage soil; MSS, mid-successional stage soil. BD, *Bassia dasyphylla* (planted in ESS), BD → MSD represents *B. dasyphylla* encroachment onto MSD. CA, *Chenopodium acuminatum* (planted in MSS); CA → ESS represents *C. acuminatum* encroachment onto ESS; CS, *Cleistogenes squarrosa* (planted in late-successional stage soil; CS → MSS represents *C. squarrosa* encroachment onto MSS).

Activities of four hydrolases (alkaline phosphomonoesterase (ALP), protease, urease, β-glucosidase) and two oxidoreductases (dehydrogenase, polyphenol oxidase) were measured across treatments (Figure 1). All six enzyme activities increased progressively along the successional gradient (MSD → ESS → MSS → LSS). Encroachment effects: (1) BD on MSD: Significantly increased (*p* < 0.01) activities of ALP, protease, β-glucosidase, and polyphenol oxidase. Activities in BD → MSD were 2.56-, 4.99-, 1.89-, and 1.54-fold higher, respectively, than that in MSD; (2) CA on ESS: Significantly increased (*p* < 0.05) ALP (1.32× ESS) and protease (1.33× ESS) activities. Changes in the other four enzymes were not significant. (3) CS on MSS: Significantly increased only polyphenol oxidase activity; the other five enzymes showed no significant change. These results demonstrate that the most substantial shifts in soil enzyme activity occur during initial successional stages (BD encroachment onto MSD). Subsequently, enzyme activities exhibit a progressive increase as the plant community develops.

### 2.2. Impact of Late-Successional Dominant Species Encroachment on Soil P Transformation Potential

**Figure 2:**
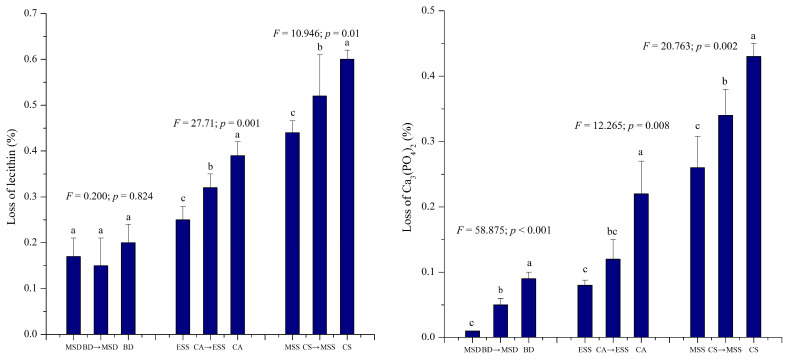
Effects of encroachment by later successional dominant species on mineralization potentials of soil organic P and solubilization potentials of soil inorganic P of the preceding stage (estimated by the loss rate of lecithin or phosphorite quantitatively added in specific microbial medium after 21 d of incubation). Values are means ± SD. *F* and *P* values are derived from one-way ANOVA. Means sharing the same lowercase letter are not significantly different (*p* < 0.05). Abbreviations: MSD, mobile sand dune; ESS, early-successional stage soil; MSS, mid-successional stage soil. BD, *Bassia dasyphylla* (planted in ESS), BD → MSD represents *B. dasyphylla* encroachment onto MSD. CA, *Chenopodium acuminatum* (planted in MSS); CA → ESS represents *C. acuminatum* encroachment onto ESS; CS, *Cleistogenes squarrosa* (planted in late-successional stage soil; CS → MSS represents *C. squarrosa* encroachment onto MSS).

The organic P mineralization rate and inorganic P solubilization rate were estimated post-incubation by measuring the leaching rates of added lecithin and phosphorite powder, respectively. Both rates increased progressively along the successional gradient (organic P mineralization: 0.17–0.60%; inorganic P solubilization: 0.01–0.43%). Notably, organic P mineralization rates significantly exceeded inorganic P solubilization rates at every successional stage. Stage-specific effects: (1) MSD → ESS: BD encroachment onto MSD did not significantly alter organic P mineralization rates (0.17–0.20%) but significantly increased inorganic P solubilization (0.01–0.09%; *p* < 0.05); (2) ESS → MSS & MSS → LSS: both mineralization and solubilization rates increased progressively (Figure 2, *p* < 0.05).

### 2.3. Impact of Late-Successional Dominant Species Encroachment on Soil phoD and gcd Gene Abundance

**Figure 3:**
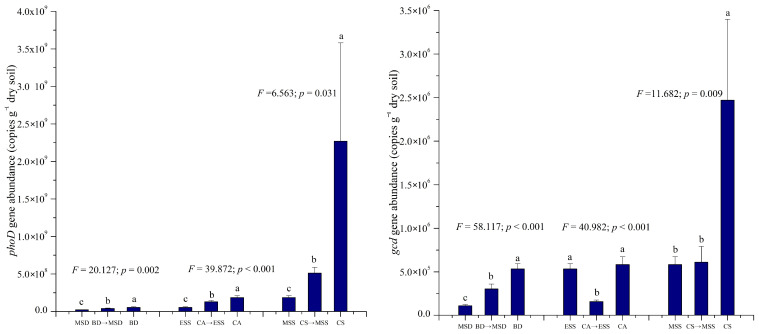
Impacts of encroachment by later successional dominant species on soil microbial *phoD* and *gcd* gene abundances of the preceding stage. Values are means ± SD. *F* and *P* values are derived from one-way ANOVA. Means sharing the same lowercase letter are not significantly different (*p* < 0.05). Abbreviations: MSD, mobile sand dune; ESS, early-successional stage soil; MSS, mid-successional stage soil. BD, *Bassia dasyphylla* (planted in ESS), BD → MSD represents *B. dasyphylla* encroachment onto MSD. CA, *Chenopodium acuminatum* (planted in MSS); CA → ESS represents *C. acuminatum* encroachment onto ESS; CS, *Cleistogenes squarrosa* (planted in late-successional stage soil; CS → MSS represents *C. squarrosa* encroachment onto MSS).

The abundances of the *phoD* and *gcd* genes in soil progressively increased along the successional sequence (LSS > MSS > ESS > MSD). *phoD* gene abundance was consistently and significantly higher than *gcd* abundance, with the *phoD*/*gcd* ratio ranging from 101.5 to 919.0. Significant differences (*p* < 0.05) in both *phoD* and *gcd* gene abundances were observed among treatments within each encroachment experiment (MSD → ESS, ESS → MSS, MSS → LSS; Figure 3). Encroachment by the next successional stage’s dominant species significantly increased *phoD* gene abundance in the soil of the preceding stage: *phoD* abundance in BD → MSD soil was 1.75-fold higher than in MSD soil; *phoD* abundance in CA → ESS soil was 2.38-fold higher than in ESS soil; *phoD* abundance in CS → MSS soil was 2.75-fold higher than that in MSS soil. Soil *gcd* gene abundance showed similar increases during the MSD → ESS and MSS → LSS transitions. However, during the ESS → MSS transition, CA encroachment onto ESS caused a transient decrease in *gcd* abundance, despite the overall increasing trend across the successional gradient. Both *phoD* and *gcd* gene copy numbers exhibited significant positive correlations (*p* < 0.01) with soil organic P mineralization rate, inorganic P solubilization rate, OM, TP, TN, and activities of all six measured enzymes (Pearson’s *r* = 0.551–0.787 for *phoD*, 0.525–0.920 for *gcd*).

### 2.4. Response of Soil phoD- and gcd-Microbial Community Structure to Late-Successional Dominant Species Encroachment

Illumina MiSeq high-throughput sequencing yielded 1,375,915 *phoD* gene sequences from 21 soil samples. Clustering at 97% similarity identified the following *phoD*-OTU counts per treatment: MSD (14,369), BD → MSD (17,758), BD (18,364), CA → ESS (10,568), CA (14,848), CS → MSS (12,422), CS (12,767). All *phoD*-OTUs were classified into 20 phyla, 29 classes, 65 orders, 104 families, 169 genera, and 308 species. Dominant *phoD*-harboring phyla (relative abundance > 1%) comprised Actinomycetota (34.21–46.20%), Pseudomonadota (19.27–32.36%), Planctomycetota (9.81–17.68%), Bacillota (1.43–9.72%), Deinococcota (1.33–3.56%), Gemmatimonadota (1.40–3.87%), and Cyanobacteriota (0.68–1.97%). Encroachment by the next successional stage’s dominant species significantly altered the *phoD*-microbial phylum structure in the preceding stage’s soil (*p* < 0.05): (1) MSD → ESS: Actinomycetota abundance decreased significantly, while Pseudomonadota and Deinococcota increased; (2) ESS→MSS: Actinomycetota and Bacillota abundances increased significantly; (3) MSS→LSS: Gemmatimonadota, Planctomycetota, and Bacillota abundances decreased significantly (Table S1).

**Figure 4:**
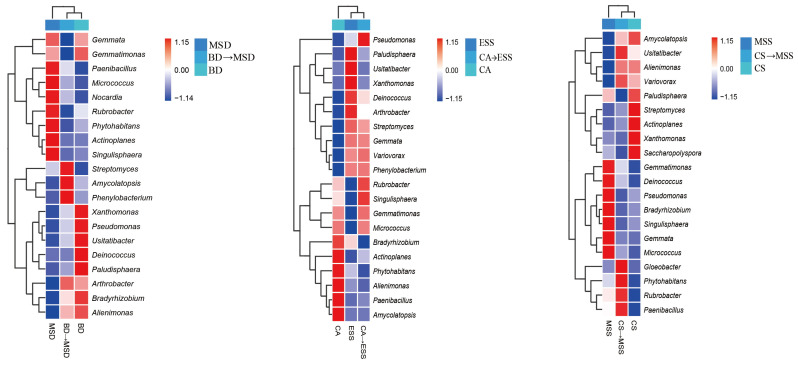
Response patterns of the relative dominance of dominant *phoD*-microbial genera to the encroachment of dominant plant species from a later successional stage onto the preceding stage. Abbreviations: MSD, mobile sand dune; ESS, early-successional stage soil; MSS, mid-successional stage soil. BD, *Bassia dasyphylla* (planted in ESS), BD → MSD represents *B. dasyphylla* encroachment onto MSD. CA, *Chenopodium acuminatum* (planted in MSS); CA → ESS represents *C. acuminatum* encroachment onto ESS; CS, *Cleistogenes squarrosa* (planted in late-successional stage soil; CS → MSS represents *C. squarrosa* encroachment onto MSS).

Encroachment by the next successional stage’s dominant species significantly altered the relative abundances of dominant *phoD*-harboring genera in the preceding stage’s soil (Figure 4), inducing genus-specific increases and decreases. Stage-specific shifts in dominant genera (*p* < 0.05): (1) MSD → ESS transition: Decreased: *Streptomyces* (18.29% → 13.86%), *Rubrobacter* (7.77% → 4.82%), *Micrococcus* (3.33% → 1.05%), *Phytohabitans* (2.06% → 1.51%), and *Nocardia* (2.06% → 1.51%); Increased: *Bradyrhizobium* (4.59% → 6.32%), *Alienimonas* (2.27% → 3.67%), *Pseudomonas* (1.58% → 2.85%), *Deinococcus* (1.76% → 2.76%), *Paludisphaera* (1.49% → 2.59%), *Usitatibacter* (1.12% → 3.39%), and *Xanthomonas* (0.94% → 1.72%). (2) ESS → MSS transition: Decreased: *Streptomyces* (15.06% → 12.48%), *Gemmata* (6.92% → 4.88%), *Variovorax* (1.73% → 0.90%), and *Phenylobacterium* (1.42% → 0.87%); Increased: *Paenibacillus* (1.76% → 7.39%), *Amycolatopsis* (2.73% → 4.33%), *Singulisphaera* (1.36% → 1.74%), and *Phytohabitans* (1.18% → 2.05%). (3) MSS → LSS transition: Decreased: *Paenibacillus* (9.32% → 5.39%), *Gemmata* (4.88% → 2.81%), *Phytohabitans* (2.90% → 1.54%), *Deinococcus* (2.40% → 1.58%), and *Singulisphaera* (1.59% → 1.02%); Increased: *Streptomyces* (12.48% → 19.17%), *Amycolatopsis* (4.33% → 6.01%), *Actinoplanes* (4.40% → 5.99%), *Xanthomonas* (1.91% → 2.76%), and *Saccharopolyspora* (0.97% → 1.43%).

**Figure 5:**
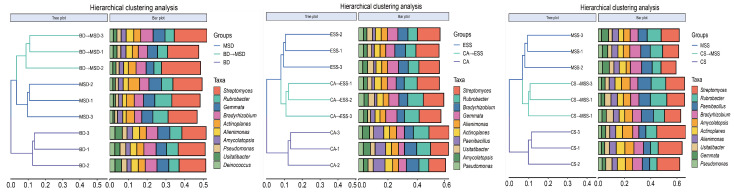
Hierarchical clustering analysis of different samples based on the relative abundances of soil dominant *phoD*-genera. Abbreviations: MSD, mobile sand dune; ESS, early-successional stage soil; MSS, mid-successional stage soil. BD, *Bassia dasyphylla* (planted in ESS), BD → MSD represents *B. dasyphylla* encroachment onto MSD. CA, *Chenopodium acuminatum* (planted in MSS); CA → ESS represents *C. acuminatum* encroachment onto ESS; CS, *Cleistogenes squarrosa* (planted in late-successional stage soil; CS → MSS represents *C. squarrosa* encroachment onto MSS).

Hierarchical clustering analysis based on the relative dominance of dominant genera grouped the different samples as shown in Figure 5. Samples from each experiment clustered into three distinct groups, with samples from the same treatment independently clustering together. This indicates significant differences in the community structure of soil microorganisms harboring the *phoD* gene.

Significant shifts in dominant species (relative abundance >1%) occurred across successional transitions (*p* < 0.05): (1) MSD → ESS transition: *Rubrobacter xylanophilus*, *Alienimonas californiensis*, *Streptomyces alboflavus*, *Deinococcus proteolyticus*, *Micrococcus luteus*, *Paludisphaera borealis*, *Phytohabitans flavus*, *Streptomyces* sp. EAS-AB2608, *Actinoplanes* sp. L3-i22, *Bradyrhizobium ottawaense*, and *Streptomyces rubrolavendulae*; (2) ESS → MSS transition: *Gemmata obscuriglobus*, *Streptomyces ambofaciens*, *Paenibacillus mucilaginosus*, *Amycolatopsis methanolica*, *Streptomyces* sp. EAS-AB2608, *Singulisphaera acidiphila*, *Phytohabitans flavus*, *Streptomyces alboflavus*, and *Actinoplanes* sp. L3-i22; (3) MSS → LSS transition: *Paenibacillus mucilaginosus*, *Streptomyces albireticuli*, *Gemmata obscuriglobus*, *Actinoplanes* sp. L3-i22, *Phytohabitans flavus*, *Deinococcus proteolyticus*, *Bradyrhizobium diazoefficiens*, and *Bradyrhizobium japonicum* (Table S2).

**Figure 6:**
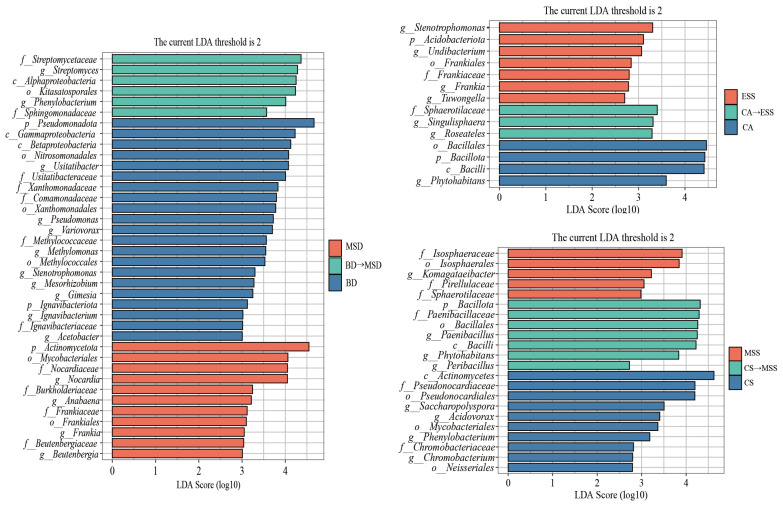
Significantly different taxa detected by LEfSe in *phoD*-microbial community. A significance level of 0.05 was used for all of the biomarkers evaluated. Only taxa meeting an LDA significance threshold of >2 were included. Abbreviations: MSD, mobile sand dune; ESS, early-successional stage soil; MSS, mid-successional stage soil. BD, *Bassia dasyphylla* (planted in ESS), BD → MSD represents *B. dasyphylla* encroachment onto MSD. CA, *Chenopodium acuminatum* (planted in MSS); CA → ESS represents *C. acuminatum* encroachment onto ESS; CS, *Cleistogenes squarrosa* (planted in late-successional stage soil; CS → MSS represents *C. squarrosa* encroachment onto MSS).

Differential taxa within the *phoD*-microbial community across treatments were identified using LEfSe analysis (Figure 6). (1) MSD → ESS transition: MSD: Actinomycetota (Mycobacteriales, Nocardiaceae, *Nocardia*), Burkholderiaceae, *Anabaena*, Frankiales (Frankiaceae; *Frankia*), and Beutenbergiaceae (*Beutenbergia*); BD → MSD: Alphaproteobacteria (Sphingomonadaceae; *Phenylobacterium*), Kitasatosporales, and Streptomycetaceae (*Streptomyces*); BD: Gammaproteobacteria (Xanthomonadales; Xanthomonadaceae; Methylococcaceae; *Pseudomonas*; *Methylomonas*; *Stenotrophomonas*), Betaproteobacteria (Nitrosomonadales; Comamonadaceae; *Variovorax*; Usitatibacteraceae; *Usitatibacter*), Ignavibacteriota (Ignavibacteriaceae; *Ignavibacterium*), *Acetobacter*, *Mesorhizobium*, and *Gimesia*. (2) ESS → MSS transition: ESS: Acidobacteriota, *Stenotrophomonas*, *Undibacterium*, Frankiales (Frankiaceae; *Frankia*), and *Tuwongella*; CA → ESS: Roseateles, Sphaerotilaceae, *Singulisphaera*; CA: Bacillota (Bacilli; Bacillales), and *Phytohabitans*. (3) MSS → LSS transition: MSS: Sphaerotilaceae, Pirellulaceae, Isosphaeraceae, *Komagataeibacter*; CS → MSS: Bacillota (Bacilli; Bacillales; Paenibacillaceae; *Paenibacillus*; *Peribacillus*), and *Phytohabitans*; CS: Actinomycetota (Mycobacteriales; Pseudonocardiales; Pseudonocardiaceae; *Saccharopolyspora*), Neisseriales (Chromobacteriaceae; *Chromobacterium*), *Phenylobacterium*, *Acidovorax*.

A total of 2,369,371 *gcd* gene sequences were obtained. Clustering at 97% similarity yielded the following *gcd*-OTU counts per treatment: MSD (81), BD → MSD (151), BD (195), CA → ESS (172), CA (231), CS → MSS (191), and CS (174). The *gcd*-microbial community exhibited exceptionally low diversity and simple structure. Identified OTUs belonged exclusively to Pseudomonadota, with relative abundance ranging from 43.02% to 93.57%. Only four *gcd*-harboring genera were identified: *Pseudomonas*, *Serratia*, *Escherichia*, and *Pantoea*. *Pseudomonas* was overwhelmingly dominant, accounting for 7.98% in MSD and 41.25% to 93.57% in other treatments. The relative dominance of the other three genera was consistently <0.01%. Several *Pseudomonas* species were also identified, primarily *P. protegens*, *Pseudomonas* sp. Ha200, *P. fluorescens*, and *P. nunensis*. *P. protegens* predominated in non-MSD samples (15.00% to 56.66%). These species showed significant inter-treatment differences in relative abundance. Given the limited diversity and structural simplicity, no further community analyses were conducted.

### 2.5. Dependence of phoD Communities on Soil Properties

**Figure 7:**
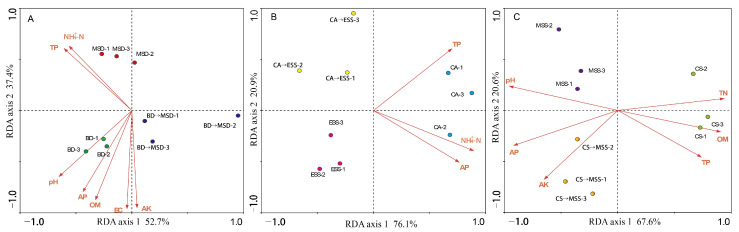
Redundancy analysis (RDA) between the structures of *phoD* community and soil properties. (**A**): MSD → ESS; (**B**): ESS → MSS; (**C**): MSS → LSS. Abbreviations: OM: organic matter; EC: electrical conductivity; TN: total N; TP: total P; AP: available P; AK: available K. MSD, mobile sand dune; ESS, early-successional stage soil; MSS, mid-successional stage soil. BD, *Bassia dasyphylla* (planted in ESS), BD → MSD represents *B. dasyphylla* encroachment onto MSD. CA, *Chenopodium acuminatum* (planted in MSS); CA → ESS represents *C. acuminatum* encroachment onto ESS; CS, *Cleistogenes squarrosa* (planted in late-successional stage soil; CS → MSS represents *C. squarrosa* encroachment onto MSS).

Redundancy analysis (RDA) elucidated relationships between *phoD* community structure and environmental factors (pH, EC, OM, TN, TP, TK, NH_4_^+^-N, AP, and AK). Ordination plots revealed significant community differences among treatments within each experiment. In MSD → ESS transition, total explained variation was 90.1% (Axis 1 = 52.7%, Axis 2 = 37.4%); In ESS → MSS transition, total explained variation was 97.0% (Axis 1 = 76.1%, Axis 2 = 20.9%); In MSS → LSS transition, total explained variation was 88.2% (Axis 1 = 67.6%, Axis 2 = 20.6%). Monte Carlo permutation tests identified key drivers at each stage: (1) MSD → ESS: pH (36.3%), EC (32.7%), OM (32.6%), AP (32.3%), AK (32.2%), TP (31.6%), and NH_4_^+^-N (30.1%) (Figure 7A); (2) ESS → MSS: NH_4_^+^-N (50.0%), AP (39.3%), and TP (35.3%) (Figure 7B); (3) MSS → LSS: pH (53.9%), TN (51.3%), AP (51.0%), OM (49.1%), TP (35.9%), and AK (32.9%) (Figure 7C). Within ordination space, replicates from the same treatment clustered tightly, while different treatments formed distinct, quadrant-separated groups. This spatial segregation further confirms significant differences in *phoD* community structure between treatments.

## 3. Discussion

### 3.1. Impact of Dominant Plant Species Shifts on Soil Properties and Biological Activity

Vegetation restoration is a highly effective approach to improving soil quality in semi-arid sandy lands. In addition to planted shrubs, drought-tolerant herbs naturally colonize and spread beneath sand-fixing forests through succession, eventually forming diverse herbaceous communities. As community composition changes across dune restoration stages, soil nutrient accumulation is markedly affected, reflecting the dynamic balance between nutrient uptake by vegetation and nutrient replenishment. Our results show that during herbaceous succession, the early encroachment of the next-stage dominant species often causes a temporary decline in certain soil nutrients, but these nutrients gradually become enriched as succession progresses. This pattern occurs because (1) range-expanding species can deplete soil nutrients during establishment, temporarily reducing accumulation; (2) soil microorganisms reduce mutualistic partnerships with native plants [24], impairing their nutrient acquisition competitiveness; (3) the range-expanding species interacts with soil microorganisms, establishing novel symbiotic relationships [25]. As environmental conditions adapt to support the invader, it colonizes, spreads, and achieves dominance. Subsequently, the species enhances soil nutrient content through continuous inputs of C, N, and P via litter decomposition and root exudates, thereby accelerating biogeochemical cycling and ecosystem energy flow.

Across the successional gradient, OM, TN, TP, and AK consistently increased. This trend likely reflects enhanced vegetation cover and diversity, particularly the proliferation of perennial grasses with dense root systems. These plants contribute substantial belowground litter, which undergoes microbial decomposition to form soil OM. Elevated OM directly facilitates nutrient accumulation, while its mineralization further promotes TN and TP enrichment [26]. Conversely NH_4_^+^-N and AP exhibited unimodal patterns, declining in later stages. This reduction may stem from: (1) elevated plant demand for bioavailable N and P; (2) nitrification-mediated conversion of NH_4_^+^-N to nitrate (NO_3_^−^-N), followed by plant uptake or leaching; (3) P fixation via complexation with Fe/Al ions forming insoluble compounds; (4) experimental constraints (e.g., limited pot trial duration, potential anthropogenic disturbance). Collectively, these factors cause nutrient consumption to outpace replenishment, limiting rapid recovery of highly labile NH_4_^+^-N and AP pools essential for plant growth.

Soil enzyme activity, a sensitive indicator of soil biological activity [27], catalyzes key biochemical reactions and responds rapidly to structural changes in the soil, making it a widely used metric for assessing soil ecosystem sustainability [28,29]. Our findings reveal progressive increases in all six measured enzyme activities throughout herbaceous vegetation succession. This trend paralleled soil nutrient responses to dominant species replacement, consistent with established positive correlations between enzyme activities and nutrients. Elevated OM enhances soil physical properties (e.g., porosity and aeration) and provides primary substrates for enzymatic reactions [30]. Increased N and P availabilities stimulate rhizosphere microbial growth, boosting enzyme synthesis. ALP, the key catalyst for organic P mineralization, directly regulates soil P bioavailability and indicates P transformation dynamics [2]. Wang et al. (2022) found that N addition promotes P fraction conversion to available P via reduced pH and elevated organic carbon and phosphatase activity [31]. Marklein & Houlton (2012) also demonstrated that N fertilization significantly increased phosphatase activity [32]. Similarly, Weand et al. (2010) showed that microbes enhance ALP secretion only under high/near-saturated soil N, aligning precisely with our results [33].

Across the successional gradient, the transformation rates of both organic and inorganic P increased progressively. However, inorganic P solubilization remained consistently low, suggesting that soil available P accumulation relies primarily on organic P mineralization, with mineral P solubilization playing only a secondary role. This functional dominance is further supported by the significantly higher abundance of *phoD*-harboring microorganisms compared to those harboring *gcd*.

Overall, during the replacement of dominant herbaceous species in sandy ecosystems, plant-soil feedback modulates the stoichiometric balance of N, P, and K, inducing coordinated shifts in key hydrolase and oxidoreductase activities. Elevated enzyme activities reflect enhanced microbial metabolic capacity, which promotes efficient nutrient cycling and soil nutrient accumulation. As succession progresses toward stable stages, nutrient consumption and replenishment gradually reach a dynamic equilibrium. The enrichment of critical nutrients, particularly OM, N, and P, ultimately sustains stable plant communities, underpinned by co-adapted development among plants, soil, and microorganisms.

### 3.2. Impact of Dominant Plant Species Shifts on Soil phoD- and gcd-Microbial Abundances

In sandy ecosystems, P mineralization represents the dominant pathway for soil AP acquisition. *phoD* gene abundance (encoding an ALP subunit) serves as a key indicator of organic P mineralization potential. Across the successional gradient, *phoD* gene copy numbers progressively increased. This is likely because (1) the inherently low AP content in aeolian sandy soil, coupled with the rapid P utilization by colonizing herbaceous vegetation under the plantation, further exacerbates soil P deficiency; (2) P limitation induces soil microorganisms to activate the *Pho* regulon, upregulating *phoD* expression and ALP secretion [2]; (3) Improved soil nutrients provide resources supporting P-transforming microbial communities.

Inorganic P solubilization is a secondary route to increasing soil AP, mainly mediated by phosphate-solubilizing bacteria (PSB) that secrete organic acids. These acids enhance P bioavailability by chelating metal cations (Ca^2+^, Fe^3+^, and Al^3+^) or lowering soil pH [34]. Gluconic acid, the dominant organic acid, is synthesized by pyrroloquinoline quinone (PQQ)-dependent glucose dehydrogenase, encoded by the *gcd* gene. Thus, gcd abundance serves as a proxy for inorganic P solubilization potential. Several *gcd*-harboring PSB (e.g., *Pseudomonas*, *Bacillus*, and *Rhizobium*) have been successfully isolated as plant-growth-promoting soil amendments [13]. Our qPCR results revealed *gcd* copy numbers (10^5^–10^6^) were significantly lower (1–4 orders of magnitude) than *phoD* abundances (10^7^–10^9^). Despite this quantitative disparity, *gcd* exhibited broadly similar successional trends to *phoD*. This indicates that soil AP accrual during succession depends predominantly on *phoD*-mediated mineralization, with *gcd*-mediated solubilization playing a secondary role. Both *phoD* and *gcd* gene abundances showed significant positive correlations (*p* < 0.01) with soil organic P mineralization rate, inorganic P solubilization rate, key nutrients (OM, TP, TN), and activities of all six soil enzymes. These findings align with established research [18,21,35].

### 3.3. Response of phoD- and gcd-Microbial Community Composition to Dominant Plant Species Shifts

Successional changes in dominant vegetation significantly restructured the *phoD*-microbial community at the phylum level. While core phyla persisted, their relative abundances shifted dynamically. Actinomycetota emerged as the consistently dominant phylum. This oligotrophic phylum exhibits competitive advantages in P cycling through extracellular enzyme production facilitating organic P mineralization, plant symbiotic capabilities enhancing nutrient acquisition, and the stress tolerance in nutrient-poor and semi-arid soils [36,37,38]. These traits explain its prevalence in P-transforming communities within resource-limited ecosystems.

Encroachment by late-successional dominant species significantly restructured the relative abundances of dominant *phoD*-harboring genera in preceding-stage soils (Figure 4). While core dominant genera persisted, community composition shifted through differential abundance changes. Notably, *Streptomyces*, *Rubrobacter*, *Bradyrhizobium*, *Gemmata*, *Actinoplanes*, and *Pseudomonas* maintained dominance across all successional stages. This aligns with prior findings, e.g., Chen et al. (2019) demonstrated that soil P availability regulates *phoD* community composition, with *Bradyrhizobium* and *Pseudomonas* dominating under long-term P fertilization [18]; Zhu et al. (2021) identified *Bacillus* (6–13%), *Bradyrhizobium* (3–8%), and *Pseudomonas* (3–5%) as dominant *phoD* genera in Inner Mongolian grasslands [39]. The functional relevance of these persistent genera explains their ecological success. *Streptomyces* is Ubiquitous biocontrol agents secreting growth-promoting secondary metabolites [40]. *Pseudomonas* was a persistent dominant genus in both *phoD*- and *gcd*-harboring communities, exhibiting dual functionality in P cycling by catalyzing organic P mineralization and solubilizing inorganic phosphates [18,34,41,42]. Its metabolites also modulate bacterial metabolism and bioactivity, thereby shaping broader community structure and function in P-limited ecosystems. This is consistent with previous studies identifying Pseudomonas as a core phosphate-solubilizing genus, along with *Bacillus*, *Enterobacter*, and *Rhizobium* [5,43]. The relative abundance of *Bradyrhizobium* progressively increased throughout succession, stabilizing in later stages. This pattern is likely due to its capacity as a symbiotic N-fixer that forms root nodules with legumes, rhizosphere enrichment through plant-microbe interactions, and adaptive responses to P stress through enhanced ALP activity and increased P transformation rates [44]. These mechanisms promote organic P mineralization while partially improving N availability. *Bradyrhizobium* thus facilitates tight coupling of soil N and P cycles, corroborated by our observed synchronous increases in ALP, protease, and urease activities along the successional gradient.

LEfSe analysis indicated that post-restoration microbial niche partitioning drives community divergence across successional stages. Competitive asymmetries among taxa for limited nutrients were intensified with sand-fixing plantation maturation and herbaceous layer development [2,45]. Crucially, we identified *phoD*- and *gcd*-microbes exhibiting high sensitivity to dominant plant shifts (Table S2). These responsive taxa represent prime candidates for developing targeted plant succession accelerators.

Soil properties directly drive shifts in microbial community structure. RDA showed that pH and nutrient status significantly shaped the *phoD* community during succession, with stage-specific dominant drivers. Notably, TP, AP, and ALP activity maintained strong and persistent associations with *phoD* community composition throughout the succession. Yang et al. (2012) identified pH, AP, and NH_4_^+^-N as primary *phoD* community determinants across land-use systems, noting positive correlations between pH/AP and *Gemmata*/*Paludisphaera* abundance [43]. Liang et al. (2022) demonstrated PGPR (Plant Growth-Promoting Rhizobacteria) enhanced ALP activity and *phoD* diversity in tobacco soils, governed by AK, pH, and AP [46]. Hu et al. (2020) reported tight coupling of AP/ALP activity with *Polymorphobacter*/*Streptomyces* abundances [13]. Furthermore, RDA indicated genus-specific nutrient responses within the *phoD* community, likely reflecting taxon-specific physiological adaptations and competitive interspecific interactions.

Collectively, this study demonstrates that structural and functional shifts in *phoD*- and *gcd*-harboring microbial communities are intrinsically linked to soil quality enhancement. Nutrient accumulation facilitates the encroachment of late-successional dominant plant species and the proliferation of P-transforming microorganisms. In turn, microbial community restructuring amplifies soil biological activity and increases P bioavailability. These changes jointly foster expansion of invading plant populations and enable colonization by additional species. This plant-soil-microbe feedback system drives progressive herbaceous vegetation succession.

## 4. Materials and Methods

### 4.1. Study Location and Site Description

The study area is situated in the Wulanaodu region (43°02′ N, 119°39′ E) of Wengniute County, Inner Mongolia, within the western Horqin Sandy Land. Research was conducted at the Wulanaodu Desertification Combating Ecological Station, the Institute of Applied Ecology, Chinese Academy of Sciences. This temperate semi-arid region experiences a typical continental monsoon climate with low humidity levels. Key climatic features include a mean annual temperature of 6.3 °C, a frost-free period of approximately 130 days, and an average annual wind speed of 4.5 m/s, resulting in 200–300 windy and dusty days annually. Annual precipitation averages 340.5 mm, predominantly occurring in July and August, while annual pan evaporation reaches approximately 2500 mm. The native vegetation represents a forest-grassland transition zone. However, overgrazing has caused severe degradation, leading to predominantly secondary vegetation characterized by psammophytic and meadow communities. Dominant habitat types include mobile and semi-mobile sand dunes, fixed sand dunes, gentle flat sandy lands, and interdune lowlands. Since the 1980s, large-scale stabilization of mobile sands has been achieved using straw checkerboard barriers combined with *C. microphylla* seeding. This effort has established extensive sand-fixing plantations of varying ages. Consequently, herbaceous vegetation has gradually invaded beneath the established shrub plantations, resulting in plant communities representing different successional stages.

### 4.2. Experimental Design and Soil Sampling

During the early-successional stage (ESS) of *C. microphylla* sand-fixing plantation establishment, surface wind erosion was still severe, and settled plant species were scarce. Vegetation was characterized by low population density and cover, dominated by annual grasses (e.g., *Setaria viridis*) and Chenopodiaceae plants (e.g., *B. dasyphylla*). By the mid-successional stage (MSS), communities transitioned to dominance by *Ch. acuminatum* and *P. flaccidum*. In the relatively stable late-successional stage (LSS), communities emerged dominated by *Cl. squarrosa* and *A. cristatum*. For this study, the representative dominant species selected for ESS, MSS, and LSS were *B. dasyphylla* (BD), *Ch. acuminatum* (CA), and *Cl. squarrosa* (CS), respectively.

Mobile sand dunes (MSD) served as the initial point of succession. Field plots representing ESS, MSS, and LSS were established within 15-year-old *C. microphylla* plantations, 40-year-old *C. microphylla* plantations, and an adjacent natural *C. microphylla* + *Ulmus pumila* sparse forest, respectively. Soil from the 0–10 cm layer was collected per plot and transported to the Wulanaodu Ecological Station. The dominant species from both the current and the next successional stage were planted on the soil sourced from each respective stage. This simulated: (1) BD invasion into MSD, (2) CA invasion into ESS, and (3) CS invasion into MSS. Specific experimental setup: MSD soil was left bare or planted with BD; ESS soil was planted with BD and CA; MSS soil was planted with CA and CS; LSS soil was planted with CS. Each treatment was replicated in 10 pots. This design created three comparative series: (1) MSD, BD invasion into MSD (BD → MSD), and BD, (2) ESS with BD, CA invasion into ESS (CA → ESS), and CA, and (3) MSS with CA, CS invasion into MSS (CS → MSS), and CS.

The cultivation experiment utilized non-woven fiber pots (30 cm diameter, 30 cm height) placed within sunken beds (25 cm deep). Pots were filled with homogenized soil, the gaps between pots were backfilled with soil, and the soil within pots was saturated with water. Following a 30-day equilibration period, seeds were sown. During drought periods, water was uniformly applied to all treatments. Weeds were removed promptly upon emergence, and seedlings were replaced as needed to maintain a density of two seedlings per pot. All seeds originated from the experimental field during the preceding year. Sowing commenced on 20 April 2024. Soil samples were collected on 20 September 2024. For each treatment, three replicate soil samples were collected. Each sample was divided into three parts: (1) air-dried for determination of physical and chemical properties; (2) stored at 4 °C for assessment of soil biological activity; (3) stored at −80 °C for subsequent DNA extraction and high-throughput sequencing.

### 4.3. Soil Property Determinations

Soil water content was determined by the oven-drying method. Soil pH and electrical conductivity were measured in a 1:2.5 (soil:water) suspension and a 1:5 (soil:water) suspension, respectively. Soil organic matter (OM) was determined by the potassium dichromate-sulfuric acid (K_2_Cr_2_O_7_-H_2_SO_4_) oxidation method. Total N (TN) was determined by the semimicro-Kjeldahl digestion method using an automatic nitrogen analyzer (Hanon K9860, Hanon Advanced Technology Group Co., Ltd., Jinan, China). Soil available P (AP) and total P (TP) were determined by the Olsen method and Dean method, respectively. Available potassium (AK) and total potassium (TK) were determined by atomic absorption spectrometry (AAS). Ammonium N (NH_4_^+^-N) was determined by the salicylic acid method. TP, AP, and NH_4_^+^-N were measured using an automatic discrete analyzer (CleverChem 380, DeChem-Tech. GmbH, Hamburg, Germany). All the above analyses were performed according to the detailed procedures described by the Institute of Soil Science, Chinese Academy of Sciences [47].

Soil alkaline phosphomonoesterase (ALP) activity was determined using the method described by Schinner et al. (1997) [48]. Soil protease activity was measured by the method of Ladd & Butler (1972) [49]. Soil urease activity was determined using the method of Kandeler & Gerber (1988) [50]. The activities of soil glucosidase were measured following the method described by Xu & Zheng (1982) [51]. Soil dehydrogenase activity was measured according to the method in ISSCAS (1985) [52]. Polyphenol oxidase activity was measured using the method described by Perucci et al. (2000) [53].

Soil microbial P turnover potential was assessed using the incubation method described by Zheng and Zhang (1982) [54]. The procedure is briefly described as follows: 10 g of fresh soil were weighed into a conical flask, mixed with 50 mL of sterile water, and shaken on an oscillator for 30 min. The basal culture medium contained: glucose 100 g, (NH_4_)_2_SO_4_ 0.5 g, NaCl 0.3 g, MgSO_4_·7H_2_O 0.3 g, KCl 0.3 g, CaCO_3_ 5.0 g, and trace amounts of MnSO_4_ and FeSO_4_, brought to a final volume of 1000 mL with distilled water. Subsequently, 35 mg of phosphorite or lecithin was added to 35 mL of the culture medium. Ten milliliters of the soil-water suspension were inoculated into the sterilized culture medium and incubated at 30 °C in the dark for 21 days. Following incubation, the AP content in the culture medium was quantified. The potential for soil microorganisms to dissolve inorganic P and mineralize organic P was calculated as the percentage of AP relative to total P added in the respective amended media.

### 4.4. Amplification, Quantification, and Sequencing of phoD and gcd

Soil genomic DNA from different treatments was extracted using a Soil DNA Rapid Extraction Kit (Bioteke, Beijing, China). Polymerase chain reaction (PCR) was used to amplify fragments of the *phoD* gene using the primer pair *phoD*-F1 (5′-TGGGAYGATCAYGARGT-3′) and *phoD*-R1 (5′-CTGSGCSAKSACRTTCCA-3′) [20], and fragments of the *gcd* gene using the primer pair *gcd*-F2 (5′-CGGCGTCATCCGGGSITIYRAYRT-3′) and *gcd*-R2 (5′-GGGCATGTCCATGTCC-3′) [55]. Real-time quantitative PCR (qPCR) targeting both genes was performed on a QuantStudio Real-Time PCR System (Applied Biosystems, Foster City, CA, USA) using SYBR Green fluorescent dye. Purified PCR products underwent paired-end sequencing on the Illumina MiSeq platform (Personal Biotechnology Co., Ltd., Shanghai, China). Operational Taxonomic Units (OTUs) were clustered at 97% nucleotide similarity. Representative sequences from each OTU were taxonomically classified using the BLASTn algorithm against the GenBank nucleotide database (http://blast.ncbi.nlm.nih.gov/Blast.cgi, accessed on 23 September 2024).

### 4.5. Data Analysis

Statistical analyses were conducted using IBM SPSS Statistics software (Version 22.0). Differences in soil physicochemical properties, enzyme activities, P turnover rates, and the abundances of the *phoD* and *gcd* genes across treatments were assessed using one-way analysis of variance (ANOVA), followed by Fisher’s Least Significant Difference (LSD) test for post hoc multiple comparisons. Statistical significance was defined as *p* < 0.05. Pearson correlation coefficients were calculated separately to examine relationships between the gene copy numbers (*phoD* and *gcd*) and (a) soil properties, and (b) P turnover rates. Differences in microbial community structure were compared using hierarchical clustering analysis based on the unweighted pair-group method. To identify taxa with differential abundance between treatments, Linear Discriminant Analysis Effect Size (LEfSe) analysis was performed, with a logarithmic LDA score threshold of >2.0 and a significance level of 0.05 for all biomarkers. Redundancy analysis (RDA) was performed using CANOCO 5.0 to determine the relative influence of soil factors on the *phoD*- and *gcd*-harboring microbial communities. The significance of soil property correlations within the RDA model was tested using Monte Carlo permutation tests. All *phoD* and *gcd* gene sequences generated in this study have been deposited in the NCBI Sequence Read Archive under BioProject accession numbers PRJNA1178444 (*phoD*) and PRJNA1178546 (*gcd*).

## 5. Conclusions

Our controlled pot experiments demonstrated that succession of herbaceous vegetation under *C. microphylla* sand-fixing plantations, driven by encroachment of late-stage dominant species, significantly improved soil nutrient status, enhanced biological activity, increased the abundance of *phoD*- and *gcd*-harboring microorganisms, and accelerated both organic P mineralization and inorganic P solubilization. Critically, we showed that plant-induced shifts in microbial community structure were taxonomically conservative at the core level but differentially altered dominant taxa, and that soil physicochemical properties (notably pH, EC, OM, TN, TP, AK, AP, and NH_4_^+^-N) were key determinants of *phoD* community composition. Importantly, P availability in these restored sandy ecosystems was governed primarily by *phoD*-mediated organic P mineralization, with *gcd*-mediated inorganic P solubilization playing only a secondary role. These findings reveal a synergistic plant–soil–microbe feedback loop that promotes further plant colonization and drives community succession.

For future work, field-scale validation over longer timescales is needed to confirm the predominant role of *phoD*. The minor contribution of *gcd* should be re-examined under different P-limitation regimes. The identified dominant *phoD*-harboring taxa offer promising candidates for bio-inoculants, but their efficacy and persistence require field testing. Practically, we suggest introducing late-successional species to accelerate P turnover, using ALP activity and *phoD* abundance as early restoration indicators, and prioritizing organic-P-mineralizing microbes for inoculant development.

## Figures and Tables

**Table 1:** Impacts of later successional dominant species encroachment on soil properties of the preceding stage.

Soil	pH	EC	OM	TN	TP	TK	NH_4_^+^-N	AP	AK
MSD	7.48 ± 0.05 b	45.57 ± 1.18 c	0.07 ± 0.01 c	0.012 ± 0.006 b	0.009 ± 0.001 a	3.41 ± 0.22 a	1.48 ± 0.05 a	3.92 ± 0.12 c	95.49 ± 8.06 b
BD → MSD	7.47 ± 0.05 b	65.35 ± 1.39 b	0.15 ± 0.01 b	0.003 ± 0.001 c	0.006 ± 00008 b	3.38 ± 0.17 a	1.18 ± 0.06 c	4.26 ± 0.10 b	161.4 ± 13.02 a
BD	7.76 ± 0.08 a	77.45 ± 1.71 a	0.29 ± 0.03 a	0.025 ± 0.003 a	0.007 ± 0.0005 b	3.34 ± 0.20 a	1.34 ± 0.04 b	5.44 ± 0.22 a	177.4 ± 6.66 a
*F*	22.471	372.395	151.174	25.374	11.246	0.098	28.764	78.541	60.879
*P*	0.002	<0.001	<0.001	0.001	0.009	0.908	<0.001	<0.001	<0.001
ESS (BD)	7.76 ± 0.08 a	77.45 ± 1.71 a	0.29 ± 0.03 a	0.025 ± 0.003 a	0.007 ± 0.0005 a	3.34 ± 0.20 a	1.34 ± 0.04 b	5.44 ± 0.22 a	177.4 ± 6.66 a
CA → ESS	7.65 ± 0.05 a	51.55 ± 2.67 c	0.28 ± 0.02 a	0.022 ± 0.004 a	0.007 ± 0.0005 a	3.62 ± 0.20 a	1.30 ± 0.10 b	4.97 ± 0.09 b	115.6 ± 5.34 c
CA	7.65 ± 0.03 a	59.35 ± 1.35 b	0.27 ± 0.05 a	0.029 ± 0.006 a	0.008 ± 0.0004 a	3.45 ± 0.21 a	1.64 ± 0.21 a	5.76 ± 0.22 a	174.6 ± 5.12 a
*F*	4.150	134.03	0.563	1.618	2.911	1.446	5.626	13.657	110.84
*P*	0.074	<0.001	0.597	0.27	0.131	0.307	0.042	0.006	<0.001
MSS (CA)	7.65 ± 0.03 a	59.35 ± 1.35 a	0.27 ± 0.05 c	0.029 ± 0.006 b	0.008 ± 0.0003 b	3.45 ± 0.21 a	1.64 ± 0.21 a	5.76 ± 0.22 b	174.6 ± 5.12 b
CS → MSS	7.58 ± 0.05 a	48.85 ± 0.74 c	0.42 ± 0.03 b	0.027 ± 0.005 b	0.009 ± 0.001 a	3.21 ± 0.11 b	1.22 ± 0.10 b	7.27 ± 0.03 a	244.2 ± 3.97 a
CS	7.40 ± 0.03 b	53.40 ± 3.16 b	0.65 ± 0.02 a	0.060 ± 0.004 a	0.010 ± 0.0005 a	3.39 ± 0.04 a	1.28 ± 0.08 b	3.20 ± 0.05 c	153.7 ± 4.99 c
*F*	35.357	20.181	94.821	63.333	4.998	2.459	7.777	712.43	302.75
*P*	<0.001	0.002	<0.001	<0.001	0.053	0.166	0.022	<0.001	<0.001

Values are means ± SD. EC: Electrical conductivity (μs·cm^−1^); OM: organic matter (%); TN: total N (%); TP: Total P (%); TK: Total K (%); AP: Available P (mg·kg^−1^); AK: Available K (mg·kg^−1^). MSD, mobile sand dune; ESS, early-successional stage soil; MSS, mid-successional stage soil. BD, *Bassia dasyphylla* (planted in ESS), BD → MSD represents *B. dasyphylla* encroachment onto MSD soil. CA, *Chenopodium acuminatum* (planted in MSS); CA → ESS represents *C. acuminatum* encroachment onto ESS soil; CS, *Cleistogenes squarrosa* (planted in late-successional stage soil; CS → MSS represents *C. squarrosa* encroachment onto MSS soil). *F* and *P* values are derived from one-way ANOVA. Means sharing the same lowercase letter are not significantly different (*p* < 0.05).

## Data Availability

The original contributions presented in the study are included in the article/Supplementary Materials; further inquiries can be directed to the corresponding author.

## Supplementary Materials

The following supporting information can be downloaded at: https://www.mdpi.com/article/10.3390/plants15142175/s1, Table S1: The relative abundance of dominant *phoD* phyla across the successional process. Table S2: Dominant soil *phoD*-harboring microbial species in soil significantly responding to the encroachment by late-successional dominant plant.
