# Supplementary material for: Impact of Dominant Species Shift in Herbaceous Vegetation Beneath Sand-Fixing Plantations on Soil Microbial Communities Involved in Organic P Mineralization and Inorganic P Solubilization
Source: Plants (Basel). 2026 Jul 15;15(14):2175. doi: 10.3390/plants15142175 (PMC13417422; doi:10.3390/plants15142175)
Supplement: Supplementary file 1 [file plants-15-02175-s001.zip › Table S1 The relative abunance of dominant phoD.pdf]

Table S1 The relative abundance of dominant *phoD* phyla across the successtional process

Treatment	Actinomycetota	Pseudomonadota	Planctomycetota	Bacillota	Deinococcota	Gemmatimonadota	Cyanobacteriota
MSD	44.22±0.498a	20.78±1.306b	14.06±0.677a	2.23±0.387a	1.76±0.124b	2.08±0.362a	0.84±0.140a
BD→MSD	39.11±2.435b	27.67±0.545a	13.36±2.662a	2.02±0.099a	1.82±0.143b	1.62±0.274a	1.02±0.044a
BD	34.55±0.585c	28.81±0.431a	15.79±0.385a	1.87±0.161a	2.77±0.067a	2.18±0.115a	1.34±0.419a
F	32.313	77.71	1.821	1.654	72.112	3.595	2.863
P	0.001	<0.001	0.241	0.268	<0.001	0.094	0.134
ESS	34.55±0.585b	28.81±0.431a	15.79±0.385ab	1.87±0.161b	2.77±0.067a	2.18±0.115a	1.34±0.419a
CA→ESS	38.95±0.337a	27.02±1.893a	16.82±0.762a	2.9±0.876b	2.83±0.35a	2.75±0.323a	1.48±0.03a
CA	38.27±2.698a	25.63±2.827a	14.56±0.759b	7.83±0.603a	2.40±0.386a	2.71±0.333a	1.63±0.187a
F	6.520	1.943	8.826	79.112	1.747	4.029	0.913
P	0.031	0.224	0.016	<0.001	0.252	0.078	0.451
MSS	38.27±2.698a	25.63±2.827a	14.56±0.759a	7.83±0.603a	2.40±0.386a	2.71±0.333a	1.63±0.187a
CS→MSS	42.4±3.308a	24.34±2.642a	10.99±0.486b	9.72±0.813a	2.51±0.412a	1.84±0.2b	1.77±0.167a
CS	46.2±3.911a	25.74±4.083a	11.6±1.117b	5.69±0.237b	2.39±0.176a	1.58±0.137b	1.42±0.044a
F	4.22	0.171	15.913	33.938	0.775	7.719	4.176
P	0.072	0.846	0.004	0.001	0.502	0.022	0.073

Values are means ± SD. *F* and *P* values are derived from one-way ANOVA. Means sharing the same lowercase letter are not significantly different ($p < 0.05$). Abbreviations: MSD, mobile sand dune; ESS, early-successional stage soil; MSS, mid-successional stage soil. BD, *Bassia dasyphylla* (planted in ESS), BD→MSD represents *B. dasyphylla* encroachment onto MSD. CA, *Chenopodium acuminatum* (planted in MSS); CA→ESS represents *C. acuminatum* encroachment onto ESS; CS, *Cleistogenes squarrosa* (planted in late-successional stage; CS→MSS represents *C. squarrosa* encroachment onto MSS).
