# Supplementary material for: Impact of Dominant Species Shift in Herbaceous Vegetation Beneath Sand-Fixing Plantations on Soil Microbial Communities Involved in Organic P Mineralization and Inorganic P Solubilization
Source: Plants (Basel). 2026 Jul 15;15(14):2175. doi: 10.3390/plants15142175 (PMC13417422; doi:10.3390/plants15142175)
Supplement: Supplementary file 1 [file plants-15-02175-s001.zip › Table S2 Domiant soil phoD-harboring species.pdf]

Table S2 Dominant soil *phoD*-harboring microbial species in soil significantly responding to the encroachment by late-successional dominant plant

Encroachment of ESS dominant plant onto MSD					
Species	MSD	BD→MSD	BD	*F*	*P*
Rubrobacter xylanophilus	7.77a	4.82b	6.05b	6.346	0.033
Alienimonas californiensis	2.27b	3.32a	3.67a	5.934	0.038
Streptomyces alboflavus	3.51a	1.97b	1.37b	60.036	<0.001
Deinococcus proteolyticus	1.76b	1.8b	2.77a	78.504	<0.001
Micrococcus luteus	3.34a	1.54b	1.05b	12.574	0.007
Paludisphaera borealis	1.49b	1.69b	2.59a	7.364	0.024
Phytohabitans flavus	2.05a	1.35b	1.51b	9.67	0.013
Streptomyces sp. EAS-AB2608	0.51b	2.02a	1.97a	6.94	0.027
Actinoplanes sp. L3-i22	1.61a	1.21b	1.14b	13.347	0.006
Bradyrhizobium ottawaense	2.27a	0.90b	0.61b	57.936	<0.001
Streptomyces rubrolavendulae	1.36a	1.45a	0.92b	13.39	0.006
Encroachment of MSS dominant plant onto ESS					
Species	ESS	CA→ESS	CA	*F*	*P*
Gemmata obscuriglobus	5.51a	5.44a	3.81b	8.079	0.02
Streptomyces ambofaciens	4.11a	4.92a	3.46b	15.451	0.004
Paenibacillus mucilaginosus	1.76b	2.13b	7.39a	98.899	<0.001
Amycolatopsis methanolica	1.23b	1.14b	2.9a	10.352	0.011
Streptomyces sp. EAS-AB2608	2.27a	1.81b	0.63b	5.688	0.041
Singulisphaera acidiphila	1.36b	1.74a	1.59ab	14.455	0.005
Phytohabitans flavus	1.51b	1.18b	2.05a	18.711	0.003
Streptomyces alboflavus	1.5a	1.64a	1.2b	12.357	0.007
Actinoplanes sp. L3-i22	1.14b	1.26b	1.81a	10.786	0.01
Encroachment of LSS dominant species onto MSS					
Species	MSS	CS→MSS	CS	*F*	*P*
Paenibacillus mucilaginosus	7.39a	9.32a	5.39b	38.641	<0.001
Streptomyces albireticuli	1.84c	2.25b	6.27a	17.243	0.003
Gemmata obscuriglobus	3.81a	2.27b	2.11b	15.856	0.004
Actinoplanes sp. L3-i22	1.81b	1.79b	3.1a	8.409	0.018
Phytohabitans flavus	2.05a	2.9a	1.54b	6.61	0.03
Deinococcus proteolyticus	2.4a	1.83b	1.58b	7.645	0.022
Bradyrhizobium diazoefficiens	1.16b	1.04a	2.18s	8.257	0.019
Bradyrhizobium japonicum	1.67a	1.58a	0.65b	17.493	0.003

The value is the average relative abundance of each species (%). *F* and *P* values, from one-way ANOVA are given. Means in the row followed by a different letter are significantly different ($p < 0.05$).

Abbreviations: MSD: mobile sand dune; ESS: early-successional stage soil; MSS: mid-successional stage soil; LSS: late-successional stage soil. BD, *Bassia dasyphylla* (planted in ESS), BD→MSD represents *B. dasyphylla* encroachment onto MSD. CA, *Chenopodium acuminatum* (planted in MSS); CA→ESS represents *C. acuminatum* encroachment onto ESS; CS, *Cleistogenes squarrosa* (planted in late-successional stage soil; CS→MSS represents *C. squarrosa* encroachment onto MSS).
